# Toward Identification of Black Lemma and Pericarp Gene *Blp1* in Barley Combining Bulked Segregant Analysis and Specific-Locus Amplified Fragment Sequencing

**DOI:** 10.3389/fpls.2017.01414

**Published:** 2017-08-14

**Authors:** Qiaojun Jia, Junmei Wang, Jinghuan Zhu, Wei Hua, Yi Shang, Jianming Yang, Zongsuo Liang

**Affiliations:** ^1^College of Life Sciences, Zhejiang Sci-Tech University Hangzhou, China; ^2^Key Laboratory of Plant Secondary Metabolism and Regulation of Zhejiang Province Hangzhou, China; ^3^Zhejiang Academy of Agricultural Sciences Hangzhou, China

**Keywords:** barley, black grain color, SLAF-seq, SNP, fine-mapping

## Abstract

Black barley is caused by phytomelanin synthesized in lemma and/or pericarp and the trait is controlled by one dominant gene *Blp1.* The gene is mapped on chromosome 1H by molecular markers, but it is yet to be isolated. Specific-locus amplified fragment sequencing (SLAF-seq) is an effective method for large-scale *de novo* single nucleotide polymorphism (SNP) discovery and genotyping. In the present study, SLAF-seq with bulked segregant analysis (BSA) was employed to obtain sufficient markers to fine mapping *Blp1* gene in an F2 population derived from Hatiexi No.1 × Zhe5819. Based on SNP screening criteria, a total of 77,542 polymorphic SNPs met the requirements for association analysis. Combining two association analysis methods, the overlapped region with a size of 32.41 Mb on chromosome 1H was obtained as the candidate region of *Blp1* gene. According to SLAF-seq data, markers were developed in the target region and were used for mapping the *Blp1* gene. Linkage analysis showed that *Blp1* co-segregated with HZSNP34 and HZSNP36, and was delimited by two markers (HZSNP35 and HZSNP39) spanning 8.1 cM in 172 homozygous yellow grain F2 plants of Hatiexi No.1 × Zhe5819. More polymorphic markers were screened in the reduced target region and were used to genotype the population. As a result, *Blp1* was delimited within a 1.66 Mb on chromosome 1H by the upstream marker HZSNP63 and the downstream marker HZSNP59. Our results demonstrated the utility of SLAF-seq-BSA approach to identify the candidate region and discover polymorphic markers at the specific targeted genomic region.

## Introduction

Most barley (*Hordeum* *vulgare.* L) varieties that are now planted and consumed for malting, brewing and feed purposes are yellow or white, but some showed purple (red), blue and black grains are used as functional food. Purple barley is due to anthocyanins accumulated in the pericarp and glumes; blue color results from anthocyanins synthesized in aleurone layer of the grain; black pigments is caused by phytomelanin synthesized in lemma and/or pericarp (Harlan, 1914). Colored cereals/plants begin receiving a growing interest due to their antioxidant properties (Satué-Gracia et al., 1997; Nam et al., 2006; Philpott et al., 2006) and protective functions under severe environments (Carletti et al., 2014). It has been reported that colored barley are rich in a large number of valuable nutrients, including phenolic compounds, anthocyanins and antioxidants, and exhibit a relatively strong oxygen radical scavenging capacity (Kim et al., 2007; Bellido and Beta, 2009). Plants with highly pigments such as phytomelanins and flavonoids are more resistant to biotic and abiotic stresses (Pandey and Dhakal, 2001; Carletti et al., 2014). In rice and sorghum, flavonoids contribute to the resistance against *Magnaporthe grisea* and *Colletotrichum* spp. (Ibraheem et al., 2010). The presence of phytomelanin layer in the sunflower pericarp serves as a deterrent to insect predation, affording mechanical protection against larval damage (Carletti et al., 2014). Dihydroquercetin, one of the flavonoids in barley is proved to be a strong inhibitor of Fusarium growth and macrospore formation (Skadhauge et al., 1997). In addition, black barley has a lower Fusarium head blight (FHB) incidence and less deoxynivalenol (DON) concentration than yellow barley after comparison of black and yellow recombinant inbred lines (RILs) from two different crosses (Choo et al., 2015).

Grain color genes have been reported in barley. Both *Pre1* and *Pre2*, located on chromosome 1H and 2H, respectively, control purple or red lemma and pericarp trait development in barley (Franckowiak et al., 1997). Recently, *Pre2* gene was mapped between InDel marker PQJ1056 and *HvOs04g47170* with genetic distance of 0.3 and 0.1 cM, respectively (Jia et al., 2016). Moreover, barley flavonoid biosynthesis regulatory genes also affect lemma colors, such as *Ant2* encoding one of the basic Helix-Loop-Helix (bHLH) proteins in the anthocyanin pigmentation pathways (Cockram et al., 2010). Barley varieties with *Ant2* gene showed red auricle, awns and lemma because of the accumulation of anthocyanin pigments in these tissues (Cockram et al., 2010). Finch and Simpson (1978) reported that five complementary dominant genes symbolized as *Blx1*, *Blx2*, *Blx3*, *Blx4*, and *Blx5*, controlled barley blue aleurone color. They assigned *Blx1*, *Blx3*, and *Blx4* to chromosome 4HL, and *Blx2* and *Blx5* to chromosome 7HL. It has been reported that black grain is dominant over yellow grain and is controlled by *Blp1* located on chromosome 1HL (Franckowiak et al., 1997). Molecular markers have been identified to associate with the black color gene *Blp1*, which is mapped at the position 129.5 cM on chromosome 1H in the Oregon Wolfe Barley (OWB) double haploid (DH) population (Costa et al., 2001). The OWB DH population was derived from the F1 of a cross between OWB-D (black grain) and OWB-R (yellow grain) using *H. bulbosum* technique (Wolfe, 1972). Genetic mapping with CAPS markers derived from high-throughput single nucleotide polymorphisms (SNPs) reveals that *Blp1* is associated with CAPS markers CAPS026 to CAPS030 in 1HL and is closely linked with CAPS029 at the position 116.3 cM in an F2 population of Cheri (yellow grain) × ICB181160 (black grain) (Bungartz et al., 2016). Presently, the *Blp1* gene is yet to be isolated.

Bulked segregant analysis (BSA) is a traditional method to rapidly map a target gene or major QTL affecting a trait of interest by genotyping only two bulked DNA samples with distinct or opposing extreme phenotypes (Michelmore et al., 1991). Specific-locus amplified fragment sequencing (SLAF-seq) is a newly efficient strategy for large-scale *de novo* SNP discovery and high-resolution genotyping (Sun et al., 2013). Combining BSA and SLAF-seq technologies have been successfully proven to be a powerful method for identifying major QTLs or candidate gene isolation in maize (Xia et al., 2015), rice (Xu F. et al., 2015), cucumber (Xu X. et al., 2015), barley (Qin et al., 2015), wheat (Hu et al., 2016), tomato (Zhao et al., 2016), and pepper (Xu et al., 2016).

Hatiexi No.1 with black lemma and pericarp, is one of the landraces from Heilongjiang Province, China. Zhang (1997) reported that the inheritance of black grain of Hatiexi No.1 was governed by *Blp1* gene due to their genetic studies involving F1 and F2 generations from the cross Hatiexi No.1 (black grain) × 93-597 (yellow grain). In this study, Hatiexi No.1 with black grain was crossed to barley variety Zhe5819 with yellow grain to construct F2 population, and we aimed to (1) find black lemma and pericarp gene-containing regions by integrating BSA with SLAF-seq technology, (2) develop SNP markers and genotype segregating populations to map the *Blp1* gene, (3) narrow down the size of the candidate gene regions, laying foundation for cloning the grain color gene.

## Materials and Methods

### Plant Materials

The black grain barley Hatiexi No.1 was crossed with the yellow grain variety Zhe5819. The resulting F1 plants were self-crossed to obtain F2. Grain color of F1 and F2 were examined in the field before harvested. The F2 population of Hatiexi No.1 × Zhe5819 consists of 551 black grain lines and 172 yellow grain lines. For mapping the gene controlling grain color, homologous yellow individuals were selected from F2 population of Hatiexi No.1 × Zhe5819.

### DNA Isolation

Young leaves of the two parents (Hatiexi No.1 and Zhe5819) and F2 individuals were collected for DNA extraction. Total genomic DNA was prepared from leaf tissues using CTAB method (Murray and Thompson, 1980). DNA concentration and quality were estimated using a Nanodrop 2000 UV-vis spectrophotometer machine and by electrophoresis through 0.8% agrose gels.

### Construction of SLAF Library for Sequencing and Analysis of SLAF-seq Data

Fifty plants with black grain and fifty plants with yellow grain were selected randomly from the F2 generation as two pools for SLAF-seq-BSA. The black pool and yellow pool were constructed by mixing an equal amount of DNA from 50 black individuals and 50 yellow individuals, respectively. The parents and two pools were used for SLAF library construction and sequencing as described previously (Sun et al., 2013; Hu et al., 2016). A pre-design SLAF experiment was designed to determine conditions and appropriate restriction enzymes for digestion that optimize SLAF yield and maximize SLAF-seq efficiency. The SLAF library was conducted in accordance using the pre-designed scheme. Genomic DNA was digested with *RsaI* (New England Biolabs, NEB). After that, a single-nucleotide A overhang were added to the digested fragments with Klenow Fragment (3′→ 5′ exo–) (NEB) and dATP at 37°C, and then the Duplex Tag-labeled Sequencing adapters (PAGE purified, Life Technologies) were ligated to the A-tailed fragments with T4 DNA ligase. PCR reaction was performed using diluted restriction-ligation DNA samples, dNTP, Q5 High-Fidelity DNA Polymerase and PCR primers: AATGATACGGCGACCACCGA and CAAGCAGAAGACGGCATACG (PAGE purified, Life Technologies). The PCR productions were purified using Agencourt AMPure XP beads (Beckman Coulter, High Wycombe, United Kingdom) and then pooled. The pooled sample was separated by electrophoresis using 2% agarose gel. Fragments with 364–394 bp (with indexes and adaptors) in size were excised, and then purified using QIAquick Gel Extraction Kit (QIAGEN). The gel-purified product was sequenced on the Illumina HiSeq 2500 system (Illumina, Inc; San Diego, CA, United States) according to the manufacturer’s recommendations.

After sequencing, all reads were aligned to barley reference genome released by The International Barley Sequencing Consortium in 2012 (IBSC 2012^1^) using BWA software (Li and Durbin, 2009). Sequences with over 90% identity were grouped in one SLAF locus. Specific fragments were considered as SLAF tags and polymorphic SLAFs were selected due to their polymorphism between two parents. Based on physical position of SLAF tags, SNP calling was performed by local realignment and mutation detection using GATK software^2^. We excluded SNPs which supported less than four reads in the two pools and showed no polymorphism between the parents because they may be false positives due to genomic repeat sequence, sequencing or alignment errors. Then SNPs showed multiple allele loci and monomorphism between the black and yellow pools were removed. Finally, SNPs with one genotype derived from Hatiexi No.1 and the other from Zhe5819 were identified as polymorphic markers, and were selected for association analysis.

### Association Analysis

Association mapping was conducted to identify candidate regions for black lemma and pericarp using both SNP_index (Abe et al., 2012; Takagi et al., 2013) and Euclidean distance (ED) methods (Hill et al., 2013).

SNP_index association analysis, a recently published method, is used to calculate genotype frequency differences between two bulks that were satisfied by Δ(SNP_index). The closer marker is associated with phenotype while the closer Δ(SNP_index) is associated with 1. M stands for Hatiexi No.1, P stands for Zhe5819, aa denotes the genotype from Hatiexi No.1 in the black pool, and ab denotes the genotype from the yellow pool. The Δ(SNP_index) was calculated as follows:

SNP_index⁢ (ab)=Mab/(Pab+Mab);

SNP_index⁢ (aa)=Maa/(Paa+Maa);

Δ(SNP_index)=SNP_index⁢ (aa)−SNP_index⁢ (ab).

Mab and Pab were the depth of yellow pool from black and yellow grain parents, respectively; and Maa and Paa indicated the depth of black pool from black and yellow grain parents, respectively.

The allelic frequency was calculated by Euclidean distance followed by Loess regression analysis which identifies regions in which QTL lies and generates a list of putative regions in the linked genomic segment.

Euclidean distance association analysis is a type of method that calculates Euclidean distance and is satisfied by ED according to Hill et al. (2013) and Geng et al. (2016). In principle, the higher the ED value is, the closer the object site. The raw ED was calculated at each SNP location using the equation:

$$ED=\sqrt{{\left(A_{aa}-A_{ab}\right)}^{2}+{\left(T_{aa}-T_{ab}\right)}^{2}}+\sqrt{{\left(G_{aa}-G_{ab}\right)}^{2}+{\left(C_{aa}-C_{ab}\right)}^{2}}$$

*A*_aa_, *C*_aa_, *T*_aa_, and *G*_aa_ represent the depth of bases *A*, *C*, *T*, and *G* on a site in the black grain bulk, respectively. *A*_ab_, *C*_ab_, *T*_ab_, and *G*_ab_ represent the depth of bases *A*, *C*, *T*, and *G* on a site in the yellow grain bulk, respectively.

In order to increase the effect of large ED measurements and decrease the effects of background noise, the allele frequency of raw ED raised to the fifth power. Then the fitting result of ED^5^ calculated using local linear regression of the EDs with a span automatically chosen by minimizing the corrected Aikaike Information Criterion (AICc) (Hill et al., 2013), was used to associate analysis.

### Markers Development by SLAF-seq Strategy and Hatiexi No.1 × Zhe5819 F2 Population Genotyping

To minimize the genetic interval for fine-mapping and to verify the accuracy of SLAF-seq, We chose at about 1 Mb one to three potential SNPs located in the candidate region (Chr 1H 427,749,941 to 460,155,270 bp, IBSC 2012) to design their flanking primers using Oligo Primer Analysis Software v.7 which ranged from 100 to 300 bp in length. PCR reaction conditions were as follows: denaturation at 94°C for 5 min, 35 amplification cycles of 94°C for 30 s, annealing at 55°C for 30 s, and extension at 72°C for 30 s, with a final extension at 72°C for 5 min. PCR products were separated on 6% polyacrylamide gel (acrylamide/bisacrylamide ratio of 37.5:1) in 0.5 × Tris-Borate-EDTA (TBE) buffer and ran at room temperature for 2–4 h, stained with silver nitrate, and observed on white illumination. Size differences in polymorphisms were identified between Hatiexi No.1 and Zhe5819. PCR products showed no size polymorphisms on the polyacrylamide gel were sequenced in one direction using the specific PCR primers distal to the potential SNP position by biosune (Shanghai) Biotechnology Co., Ltd. The Megalign program (DNAStars) was used for sequence alignment and to confirm SNP sites.

The confirmed SNP markers were genotyped in 172 homozygous yellow individuals from F2 of Hatiexi No.1 × Zhe5819 following SNP marker detection with direct DNA sequencing or KASPar platform. Kompetitive Allele-Specific PCR (KASP) is a SNP genotyping system from LGC Genomics (United Kingdom) that tags different fluorescent dye to each SNP allele during the PCR reaction. Twenty two SNP markers were detected employing KASPar platform in segregating population by Beijing Vegetable Research Center (China). The KASP genotyping procedures were followed according Wen et al. (2015). The size differences markers were identified by polyacrylamide gel electrophoresis (PAGE). Other polymorphic markers were analyzed by Sanger DNA sequencing.

### Genetic Mapping

Linkage analysis of the molecular markers and black grain trait was performed using MAPMAKER version 3.0 software (Lander et al., 1987). Map distances were estimated using the Kosambi equation (Kosambi, 1944). For fine mapping, closer markers linked to the candidate gene were further developed and tested for their polymorphisms between the parents using Sanger DNA sequencing. Polymorphic markers were used for analysis of yellow grain plants from F2 generation. The alleles with the same genotype as that of black grain landrace Hatiexi No.1 was marked as ‘1,’ and alleles with the same genotype as that of yellow grain variety Zhe5819 was labeled as ‘0.’ For F2 plants with yellow grain, there are three possible genotypes for these markers, namely non-recombinants with ‘0/0,’ single recombinants with ‘0/1,’ and double recombinants with ‘1/1.’

## Results

### Analysis of Slaf-seq-Bsa Data and Slaf Tags

After SLAF library construction and high-throughput sequencing, a total of 180,828,494 valid single-end reads were obtained, with each read length of ∼100 bp (**Table 1**). The GC content was 43.10% and the Q30 ratio was 92.92%. The SLAF numbers were 160,977 for Hatiexi No.1 and 181,313 for Zhe5819. The average sequence depths of SLAFs were ∼16.44- and ∼27.12-fold in black parent (Hatiexi No.1) and yellow parent (Zhe5819), respectively; and ∼45.41- and ∼41.27-fold in the black pool and yellow pool, respectively, (**Table 1**). SLAF tags were mapped on barley assembly (IBSC 2012) and 233,701 SLAFs markers distributed throughout the genomes. The SLAF numbers and chromosome positions are shown in **Table 2**.

**Table 1:** Summary of the sequencing data for each sample.

Sample	Total reads	GC %	Q30%	SLAF number	Total depth	Average depth
Hatiexi No.1	17,631,146	42.81	92.2	160,977	2,646,465	16.44
Zhe5819	38,025,068	43.41	93.3	181,313	4,916,562	27.12
Black pool	63,431,978	43.20	93.4	216,958	9,852,375	45.41
Yellow pool	61,740,302	42.98	92.8	205,768	8,492,938	41.27
Total	180,828,494	–	–	765,016	–	–


**Table 2:** Number distribution of specific-locus amplified fragment (SLAF) tags, single nucleotide polymorphism (SNP) markers, polymorphic SLAF and SNP on each chromosome.

Chromosome	SLAF number	All SNP	Polymorphic SLAF	Polymorphic SNP
Chr 1H	22,762	20,349	6,768	7,708
Chr 2H	35,479	25,365	7,495	8,660
Chr 3H	32,596	37,540	12,767	14,690
Chr 4H	31,198	19,263	5,703	6,404
Chr 5H	31,635	23,457	6,740	7,750
Chr 6H	28,518	31,182	10,605	11,648
Chr 7H	32,883	38,521	12,755	14,594
Chr unknown	18,630	20,045	5,144	6,088
Total	233,701	215,721	67,977	77,542


### Polymorphic SNP Markers Screening

From the 233,701 SLAF tags, 215,721 SNPs were obtained after aligning the sequence data to the barley reference. At the stage of SNP calling, SNPs with multiple allele loci and a depth less than 5× were filtered out. Polymorphic SNPs refer to SNPs that show polymorphic not only between the parents but also between the two bulked DNA samples. Finally, 77,542 polymorphic SNPs were ultimately selected for further analysis and the statistics of marker numbers on each chromosome according to the positioning result are shown in **Table 2**.

### Association Analysis with SNP_index and Euclidean Distance

**FIGURE 1:**
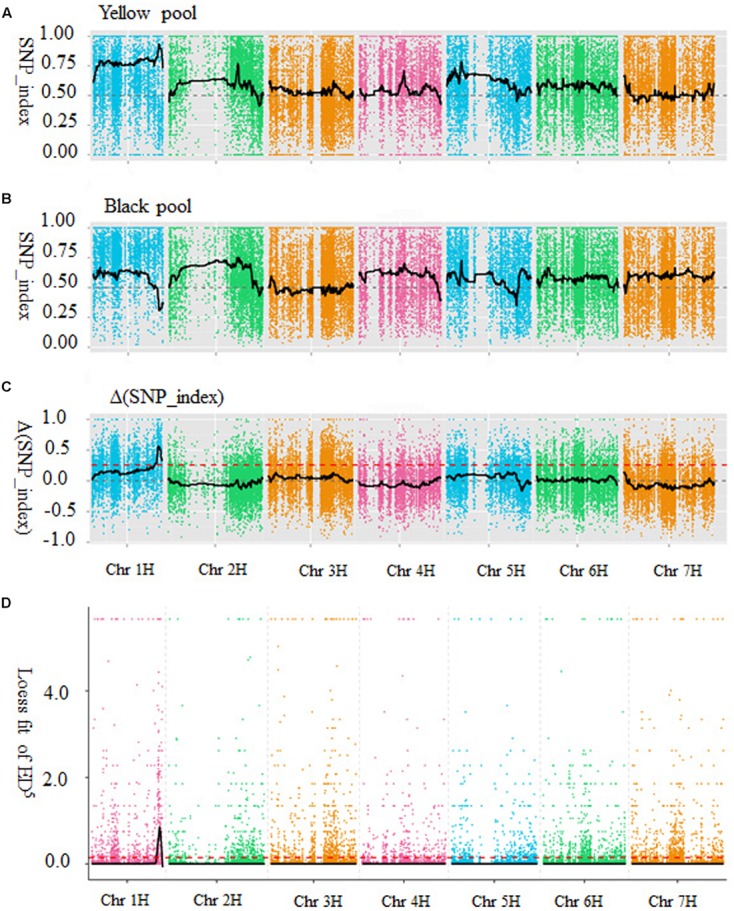
Identification of the candidate region for black lemma and pericarp through two types of association methods. **(A)** SNP_index graph of yellow pool. **(B)** SNP_index graph of black pool. **(C)** SNP_index graph of Δ(SNP_index). The red dot line is the threshold value (0.26). **(D)** The results of Euclidean distance association analysis. The black lines show all fitting results of ED^5^, red dot lines show the threshold of ED. *X*-axis represents the position of seven chromosomes and *Y*-axis represents the SNP_index or Loess fit of ED^5^.

Both SNP_index and Euclidean distance association analysis were used to identify the candidate regions for barley black lemma and pericarp trait. For the SNP_index method, SNP_index was calculated for each identified SNP according to Abe et al. (2012) and Takagi et al. (2013). An average SNP_index of SNPs was calculated with 200 SNP_indexes located in a given genomic interval. SNP_index graphs were generated for the yellow (**Figure 1A**) and black (**Figure 1B**) pools by plotting the average SNP_index against the position of each sliding window in the barley genome assembly (IBSC 2012). After combining the SNP_index information into the yellow and black pools, the Δ(SNP_index) was calculated and plotted against the genome positions (**Figure 1C**). Peak regions above the threshold value were defined as those where Loess fitted values were greater than standard deviations above the genome-wide median in the Δ(SNP_index) plot. One candidate region associated with barley black grain spanned 49.28 Mb on chromosome 1H (from 414,847,463 to 464,122,721 bp, barley genome assembly, IBSC 2012), was identified with Δ(SNP_index) value above the threshold value of 0.26 (**Figure 1C**).

Euclidean distance (ED) was calculated for each SNP according Hill et al. (2013). To increase the effect of large ED measurements and decrease the effects of low ED measurements/noise, the 5th power of ED was calculated as the correlation value. The association threshold was 0.15 and one region on chromosome 1H was significantly correlated with the black lemma and pericarp trait. The result of the Euclidean distance association analysis was shown in **Figure 1D**. According to barley physical map (IBSC 2012), the candidate region was physically located on chromosome 1H between 427,749,941 and 460,155,270 bp, with a size of 32.41 Mb.

Combining the results of SNP_index and Euclidean distance association analysis suggested that the overlapped region (427,749,941–460,155,270 bp on chromosome 1H, IBSC 2012) was the candidate region of the barley black lemma and pericarp gene.

### Validation of the SNP Markers and Mapping the Candidate Gene

A total of 524 potential polymorphic SNPs were obtained in the 32.41 Mb candidate regions (Supplementary Table S1). To evaluate the accuracy of SLAF genotyping data, one to three SNPs per Mb were selected across the entire candidate region. Fifty four pairs of primers were designed due to their potential polymorphisms and physical position on barley genome assembly (IBSC 2012). Markers polymorphisms between Hatiexi No.1 and Zhe5819 were verified by electrophoresis and independent traditional Sanger sequencing. Thirteen of fifty four primer pairs showed no PCR products in one parent or both parents and were removed from analysis. HZSNP34 makers showed InDel polymorphism on polyacrylamide gels. The rest of the PCR products of the two parents showed no size polymorphisms on the polyacrylamide gels were sequenced directly. Sequences alignment between the parents identified twenty nine polymorphic markers (Supplementary Table S2). Among the twenty nine polymorphic markers, 24 markers showed SNP and five of them showed multi-nucleotide polymorphisms (Supplementary Table S2).

KASPar platform was used to conduct SNP genotyping in the F2 population consisting of 172 homozygous yellow grain individuals. Twenty two KASPar type SNP markers, including 19 SNP markers and 3 multi-nucleotide polymorphism markers (HZSNP15, HZSNP28, and HZSNP36), were designed (Supplementary Table S3). For three multi-nucleotide polymorphism markers, KASPar assays just screened one SNP, which is more than 50 bp away from the other variant sites. Except HZSNP28, all the KASPar type SNP markers genotyped the population successfully. InDel marker HZSNP34 was distinguished easily on 6% polyacrylamide gel in the population.

**FIGURE 2:**
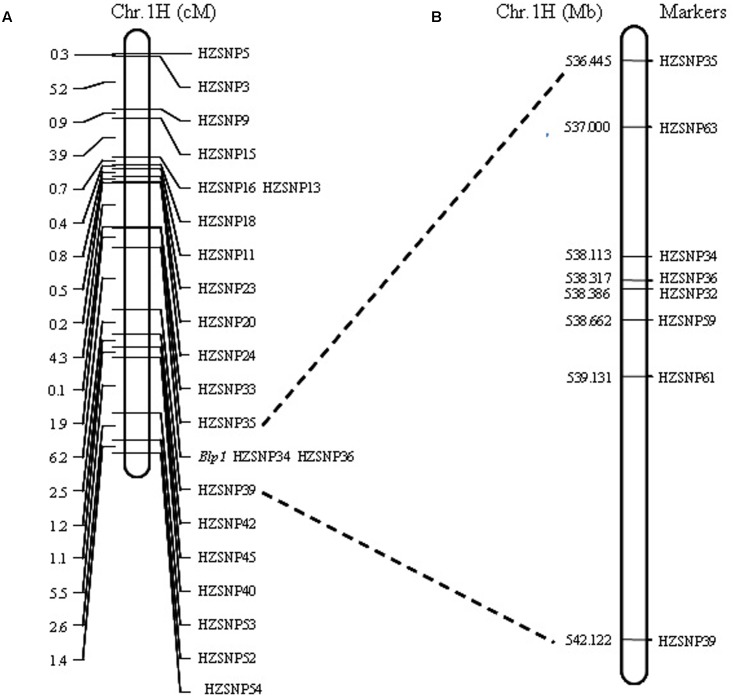
Mapping of the *Blp1* gene. **(A)** The *Blp1* gene was restricted to the region between markers HZSNP35 and HZSNP39; **(B)** the *Blp1* gene was further narrowed down to the region between markers HZSNP63 and HZSNP59.

Linkage analysis showed that all markers were assigned to the target regions and the gene controlling black lemma and pericarp was delimited by markers HZSNP35 (1.9 cM) and HZSNP39 (6.2 cM) (**Figure 2A**). Moreover, the gene was co-segregated with HZSNP34 and HZSNP36. These results suggested that the markers mined from SLAF-seq-BSA data are reliable. According to the barley genome assembly (IBSC 2012), the markers order in the genetic map was not consistent with its physical map (**Figure 2A** and Supplementary Table S2). Thus, all marker sequences were blast against the current barley assembly released by the International Barley Sequencing Consortium in 2017 (IBSC 2017^3^). Blast alignment analysis showed that the genetic map was incompliance with the current physical map (IBSC 2017) and the physical distance between markers HZSNP35 (536444825–536445008 bp) and HZSNP39 (542121828–542122039 bp) to be approximately 5.68 Mb on IBSC 2017 assembly (Supplementary Table S2). The chromosomal location of this locus corresponded with black lemma and pericarp1 (*Blp1*) described by Costa et al. (2001) and Bungartz et al. (2016). Hence, we also named the gene as *Blp1* following previously.

### Fine Mapping the *Blp1* Gene

Markers developed by SLAF-seq in the 5.68 Mb (HZSNP35–HZSNP39) intervals were further screened to obtain polymorphic markers between the parents with direct DNA sequencing. Four polymorphic markers, including three co-dominant markers (HZSNP59, HZSNP61 and HZSNP63) and one dominant marker (HZSNP62) were identified (Supplementary Table S2). Then the co-dominant markers and HZSNP32 located in the reduced target region, were used to analyze the genotypes of yellow pericarp F2 plants. Among the 172 homozygous yellow F2 plants of Hatiexi No.1 × Zhe5819, six plants (Y225, Y314, Y333, Y372, Y401, and Y406) were recombinants on the HZSNP63 locus and nine plants (Y316, Y415, Y328, Y330, Y331, Y332, Y379, Y422, and Y444) on the HZSNP61 locus (**Table 3**). Two plants (Y316 and Y415) appeared to be recombinants on locus HZSNP59 in the downstream (**Table 3**). Because of the limited markers, no recombinant loci were found to be closer than HZSNP63. Eventually, the *Blp1* gene was delimited within a 1.66 Mb (IBSC 2017 assembly, Chr 1H: 536,999,583-538,661,822) by the upstream marker HZSNP63 and the downstream marker HZSNP59 (**Figure 2B** and **Table 3**).

**Table 3:** The InDel and SNP genotype of yellow F2 plants of Hatiexi No.1 × Zhe5819 used for fine mapping of the *Blp1* gene.

Marker	Y225	Y314	Y333	Y372	Y401	Y406	Y316	Y415	Y328	Y330	Y331	Y332	Y379	Y422	Y444
HZSNP63	0/1	0/1	0/1	0/1	0/1	0/1	0/0	0/0	0/0	0/0	0/0	0/0	0/0	0/0	0/0
HZSNP34	0/0	0/0	0/0	0/0	0/0	0/0	0/0	0/0	0/0	0/0	0/0	0/0	0/0	0/0	0/0
HZSNP36	0/0	0/0	0/0	0/0	0/0	0/0	0/0	0/0	0/0	0/0	0/0	0/0	0/0	0/0	0/0
HZSNP32	0/0	0/0	0/0	0/0	0/0	0/0	0/0	0/0	0/0	0/0	0/0	0/0	0/0	0/0	0/0
HZSNP59	0/0	0/0	0/0	0/0	0/0	0/0	0/1	0/1	0/0	0/0	0/0	0/0	0/0	0/0	0/0
HZSNP61	0/0	0/0	0/0	0/0	0/0	0/0	0/1	0/1	0/1	0/1	0/1	0/1	0/1	0/1	0/1


*‘0’, the banding pattern was the same as that of yellow barley parent Zhe5819; ‘1’, the banding patter was the same as that of black balrey parent Hatiexi No.1.*

## Discussion

Bulked segregant analysis coupled with SLAF-seq has facilitated the rapid identification genomic regions associated with genes or QTLs in plants. Genes controlling qualitative traits, such as barley Stage Green-Revertibel Albino (Qin et al., 2015), cucumber fruit flesh thickness (Xu X. et al., 2015), maize inflorescence meristem size (Xia et al., 2015) and tomato *Cladosporium fulvum*-resistant (Zhao et al., 2016), were finely mapped in association analysis by SLAF-seq-BSA method. Using the same approach, major QTLs for grain weight were detected in rice and wheat, respectively (Xu F. et al., 2015; Hu et al., 2016). In the present study, polymorphic SNPs were obtained between two barley parents based on BSA combined with SLAF-seq. Both SNP_index and Euclidean distance association analysis identified *Blp1* candidate region with a size of 32.41 Mb on chromosome 1H, which correspond to the locus identified by Costa et al. (2001) and Bungartz et al. (2016). This result confirms that SLAF-seq combined with BSA is a high-efficient strategy for mapping the candidate gene using an F2 population.

With the development of next-generation sequencing (NGS) technologies, NGS-derived SNPs have been reported in Arabidopsis (Jander et al., 2002), rice (Feltus et al., 2004), barley (Close et al., 2009), maize (Jones et al., 2009), soybean (Hyten et al., 2010), wheat (Trebbi et al., 2011), eggplant (Barchi et al., 2011), sorghum (Nelson et al., 2011), *Aegilops tauschii* (You et al., 2011), oat (Oliver et al., 2011), and cotton (Byers et al., 2012) to name a few. Besides the ongoing revolution in sequencing techniques, high-throughput genotyping platforms of SNPs, including GoldenGate, high-resolution melting (HRM), SNaPshot multiplex SNP genotyping, TaqMan SNP genotyping, KASPar assay and MassARRAY, were developed rapidly in recent years. As a result, in most species, SNPs have become the first choice for marker development, genome-wide association studies, gene/QTL mapping, phylogenetic analyses, marker-assisted selection, BSA, and genomic selection (Xu X. et al., 2015). In the present study, we used SLAF-seq-BSA to discover SNPs by comparing SLAF-seq reads derived from two barley parents. Potential polymorphic SNPs covered the target regions were selected and their polymorphisms between the parents were tested by electrophoresis and Sanger DNA sequencing. Twenty two SNPs and one InDel markers were genotyped in the population by KASPar platform and electrophoresis, respectively. All genotyped markers were associated with *Blp1*, which verified the accuracy of the candidate region detected by associated analysis. Linkage analysis showed that the candidate region was defined into 5.68 Mb in barley physical map (IBSC 2017). Moreover, SNPs in the narrowed down regions were further screened and four additional polymorphic SNPs were used to analyze the F2 population. Markers HZSNP63 and HZSNP59 were delimited the candidate region which was declined to an interval of 1.66 Mb. This result demonstrates that markers discovered within the mapping interval by SLAF-seq-BSA strategy, are available for fine mapping in barley.

Colored grains are ubiquitous in cereals and are determined by the pigmentation of certain phytochemicals, such as anthocyanin. In plants, the anthocyanin biosynthesis pathway has been elucidated (Shih et al., 2008) and transcriptional regulation related to anthocyanin biosynthesis has also been extensively studies in Arabidopsis, maize, petunia, and other species (Yuan et al., 2009). Such regulatory proteins including basic helix-loop-helix (bHLH) transcription factors, R2R3 Myb transcription factors and WD40 proteins act in a ternary complex, as MBW (MYB-bHLH-WD40) complex transcription factors (Hichri et al., 2011; Petroni and Tonelli, 2011). In cereals, some of the genes controlling grain colors were isolated successfully. Red rice is controlled by two loci *Rc* and *Rd*, which encodes a bHLH transcription factor and dihydroflavonol-4-reductase (DFR), respectively (Sweeney et al., 2006; Furukawa et al., 2007). One of the complementary genes controlling purple rice is *Ra*, which is a member of Myc family genes and known to be involved in the biosynthesis of anthocyanin in rice (Hu et al., 1996). Black rice is the results of three complementary genes, symbolized as *Kala1*, *Kala3*, and *Kala4*. It has been speculated that the *Kala1* and *Kala3* genes encode a DFR and an R2R3-Myb transcriptional factor, respectively, and play subsidiary roles in the black rice trait (Maeda et al., 2014). *Kala4* acted as a main contributor, encodes a bHLH transcription factor and regulates anthocyanin biosynthesis (Oikawa et al., 2015). The genetic basis of wheat purple grain pigmentation resides in the action of *Pp-1* homoealleles and *Pp3* (Dobrovolskaya et al., 2006). The former was deduced as a MYB-like transcription factors responsible for the activation of structural genes encoding various enzymes participating in anthocyanin synthesis based on comparative mapping (Khlestkina, 2013). The latter was orthologous to maize *Lc* (Ludwig et al., 1989) and rice *Ra* (Hu et al., 1996), and *TaMyc1* was identified as a candidate gene for *Pp3* (Shoeva et al., 2014), which encoded MYC-like transcriptional factor underlying the regulations of anthocyanin synthesis.

It has been reported that purple (red) and blue barley are rich in anthocyanins, while black barley is caused by phytomelanin (Harlan, 1914). Barley *Ant2* gene affects red color in auricle, awns and lemma, and encodes for a transcription factor with a bHLH domain (Cockram et al., 2010). Shoeva et al. (2016) reported that *Ant2* was up-regulated with coordinately co-expressed flavonoid biosynthesis structural genes (*Chs*, *Chi*, *F3h*, *Dfr*, and *Ans*), which led to total anthocyanin content increase in the purple-grained ‘Bowman’ near-isogenic lines (NILs) with *Ant2*. However, in the black-grained ‘Bowman’ NILs, no differentially expressed flavonoid biosynthesis structural genes (with the exception of *Chi*) in comparison with Bowman were detected (Shoeva et al., 2016). As a result, anthocyanin content shows similar low amounts between Bowman and black-grained ‘Bowman’ NILs (Shoeva et al., 2016). To sum up, it seems that the grain color genes isolated so far were involved in anthocyanin synthesis or acted as transcriptional regulators. Shoeva et al. (2016) suggested that anthocyanins and the other flavonoids unlikely participated in black pigmentation of barley lemma and pericarp. Moreover, chemical nature of the black pigments and its biosynthesis pathway is still not clear (Pandey and Dhakal, 2001; Jana and Mukherjee, 2014). Therefore, the isolation of the *Blp1* gene will help to understand the mechanism of black pigmentation accumulation in barley as well as to extend it further to other plants. In this study, we mapped the *Blp1* gene into a 1.66 Mb intervals (**Figure 2** and **Table 3**). There are 40 genes and some of them are annotated in this interval based on the assembly of IBSC 2017 (Supplementary Table S4). Plant cytochrome P450 monooxygenases play critical roles in the metabolism of secondary metabolites, such as pigment. For example, the color of flowers can be modified through hydroxylation pattern determined by two P450 enzymes (CYP75B and CYP75A) (Tanaka and Brugliera, 2013). Rasika et al. (2016) reported that Cytochrome P450 (CYP450) enzymes performed the initial step in yellow and red-violet betalains pigment biosynthesis in beets. As transcription regulators participate in anthocyanin biosynthesis (Hichri et al., 2011; Petroni and Tonelli, 2011), both sequence-specific DNA binding transcription factor and TATA element modulatory factor may be involved in transcription regulation during phytomelanin accumulation. Therefore, the genes encoding Cytochrome P450 superfamily protein, sequence-specific DNA binding transcription factors and TATA element modulatory factor may be reasonable candidates for the *Blp1*. Further research is required to identify the functional gene for the *Blp1*. We are expanding the F2 population of Hatiexi No.1 × Zhe 5819 and more homologous yellow individuals will be selected to identify recombinants. Furthermore, additional markers based on the candidate gene sequences are in the process of generating new polymorphic molecular markers to refine the region for the positional cloning of underlying gene.

## Conclusion

We demonstrated the utility of SLAF-seq-BSA approach to identify the candidate region associated with barley black grain trait and discover polymorphic markers at the specific targeted genomic region. The *Blp1* gene controlling black lemma and/or pericarp was fine mapped in a size of 1.66 Mb with 40 candidate genes.

## Author Contributions

QJ, JY, and ZL designed the experiments. QJ and JW performed marker development and mapping analysis. JZ and YS contributed to phenotype Hatiexi No.1 × Zhe5819 population. WH conducted bioinformatic analysis of SNP data. QJ and ZL wrote the paper. All authors have read, edited and approved the current version of the manuscript.

## Conflict of Interest Statement

The authors declare that the research was conducted in the absence of any commercial or financial relationships that could be construed as a potential conflict of interest. The reviewer AT and handling Editor declared their shared affiliation, and the handling Editor states that the process met the standards of a fair and objective review.

## Footnotes

1 http://ensembl.gramene.org/Hordeum_vulgare/Info/Index

2 https://software.broadinstitute.org/gatk/documentation/

3 https://www.ncbi.nlm.nih.gov/assembly/GCA_900002345.1
